# Beyond Infection and Occlusion: Unusual CT Findings of Tunneled Peritoneal Catheters and Their Conservative Management

**DOI:** 10.7759/cureus.117918

**Published:** 2026-10-14

**Authors:** Joel O Kuster, Christoph Gubler, Fritz R Murray

**Affiliations:** 1 Department of Gastroenterology and Hepatology, Stadtspital Zurich, Zurich, CHE

**Keywords:** malignant ascites, palliative treatment, peritoneal catheter complication, permanent tunneled peritoneal catheters, pleurx catheter

## Abstract

Permanent tunneled peritoneal catheters (PTPCs) provide an established option for symptom control in patients with recurrent ascites. Although adverse events are usually related to infection, hemorrhage, impaired drainage, or fluid leakage, less typical complications can occur. We describe two unusual PTPC-associated complications that resolved with conservative treatment while the catheters remained in place.

Case 1 is a 57-year-old woman with metastatic breast cancer and peritoneal carcinomatosis who underwent PTPC placement for recurrent symptomatic ascites. The following day, she developed severe right shoulder pain, with CT showing cranial catheter migration and diaphragmatic contact. With continued ascites drainage, symptoms resolved completely despite unchanged catheter position at six-week follow-up.

Case 2 is a 71-year-old man with metastatic cholangiocarcinoma who underwent PTPC placement for recurrent ascites. Ten days later, he developed extensive right thoracoabdominal swelling. CT showed a large epifascial and subfascial fluid collection caused by ascites leakage through the peritoneal entry site. Frequent small-volume drainage and contralateral positioning resulted in complete resolution without catheter removal.

In clinically stable patients with a functioning catheter, even unusual mechanical complications may be successfully managed conservatively despite unusual imaging findings. To our knowledge, neither catheter-tip-induced shoulder pain from diaphragmatic irritation nor such extensive subcutaneous ascites tracking has previously been reported with corresponding CT documentation. Catheter removal should be reserved for irreversible dysfunction, dislocation, or infection uncontrolled by antimicrobial therapy.

PTPC-related complications do not necessarily require catheter removal. In clinically stable patients with a functioning catheter, even unusual mechanical complications may be successfully managed conservatively despite unusual imaging findings.

## Introduction

Permanent tunneled peritoneal catheters (PTPCs) are increasingly used for the palliative management of recurrent malignant and non-malignant ascites, particularly in patients requiring frequent drainage. The catheter is placed into the peritoneal cavity and tunneled subcutaneously, allowing repeated drainage of ascitic fluid without the need for repeated hospital-based paracentesis. This approach can provide symptom relief and facilitate outpatient or home-based management in patients with limited life expectancy.

Reported complications of PTPC placement predominantly include infection, bleeding, catheter obstruction, and pericatheter leakage [1,2]. While these complications are generally well recognized, unusual mechanical or fluid-related complications may also occur and can result in striking clinical and radiological findings. Importantly, such findings do not necessarily indicate catheter dysfunction or require catheter removal in clinically stable patients with preserved drainage function. We present two cases of uncommon PTPC-related complications that were successfully managed conservatively without catheter removal.

## Case presentation

As a general note, both catheters (Drainova® ArgentiC, Ewimed, Hechingen, Germany; size 15.5 Fr., length: 660mm, single-cuff) were implanted by experienced gastroenterologists with antibiotic prophylaxis and ultrasonographic guidance according to the manufacturer’s instructions. The silicone catheter was inserted through a subcutaneous tunnel (6-10cm length) with the distal fenestrated end placed in the peritoneal cavity. Immediately after insertion, the ascites was completely drained to reduce pressure on the puncture site and thus ensure that the peritoneum adheres.

Case 1

A PTPC was placed in the left lower quadrant of a 57-year-old woman with osseous, pulmonary, and peritoneal metastases from left-sided lobular breast cancer receiving palliative systemic therapy for recurrent symptomatic ascites. On the morning after catheter placement, the patient reported severe pain in the right shoulder. Clinically, the patient remained stable at all times, in particular showing no signs of systemic inflammation or circulatory instability. Initially, the pain did not respond to an escalation of analgesia, consisting of an additional fentanyl patch (6 mcg/h) on top of the baseline patch (12 mcg/h) and repeated intravenous doses of 3 mg morphine. The pain improved primarily with movement. Given the severity of the pain, an abdominal CT scan was performed.

The scan showed a cranially positioned catheter between the liver and the thoracic wall, with the catheter tip in contact with the diaphragm (Figure 1). Due to the lack of radiological images taken immediately after the procedure, it remains unclear whether this condition was already present immediately after catheter placement or whether a catheter shift occurred later on.

**Figure 1:**
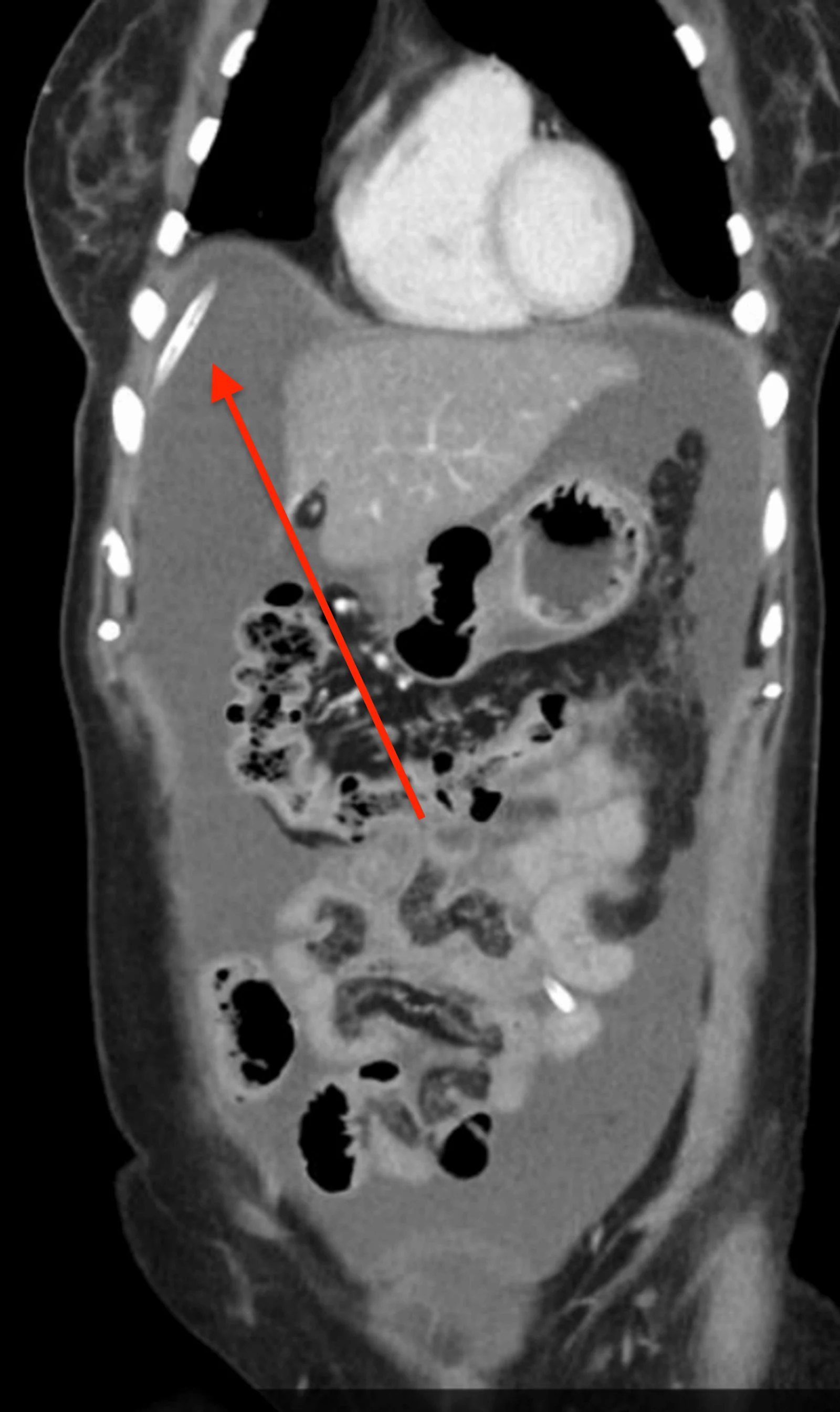
Computer tomography (coronal view) of the abdomen (June 2, 2026) The catheter tip is located between the thoracic wall and the liver, in contact with the diaphragm.

The clinical and radiological findings were consistent with diaphragmatic irritation causing referred pain to the right shoulder via the C3-C5 dermatomes. Given the preserved catheter function, conservative management with regular ascites drainage was continued. Three days after implantation, the symptoms had resolved completely despite an unchanged position of the catheter tip on imaging (abdominal CT six weeks after the initial CT was performed for oncological indications, Figure 2).

**Figure 2:**
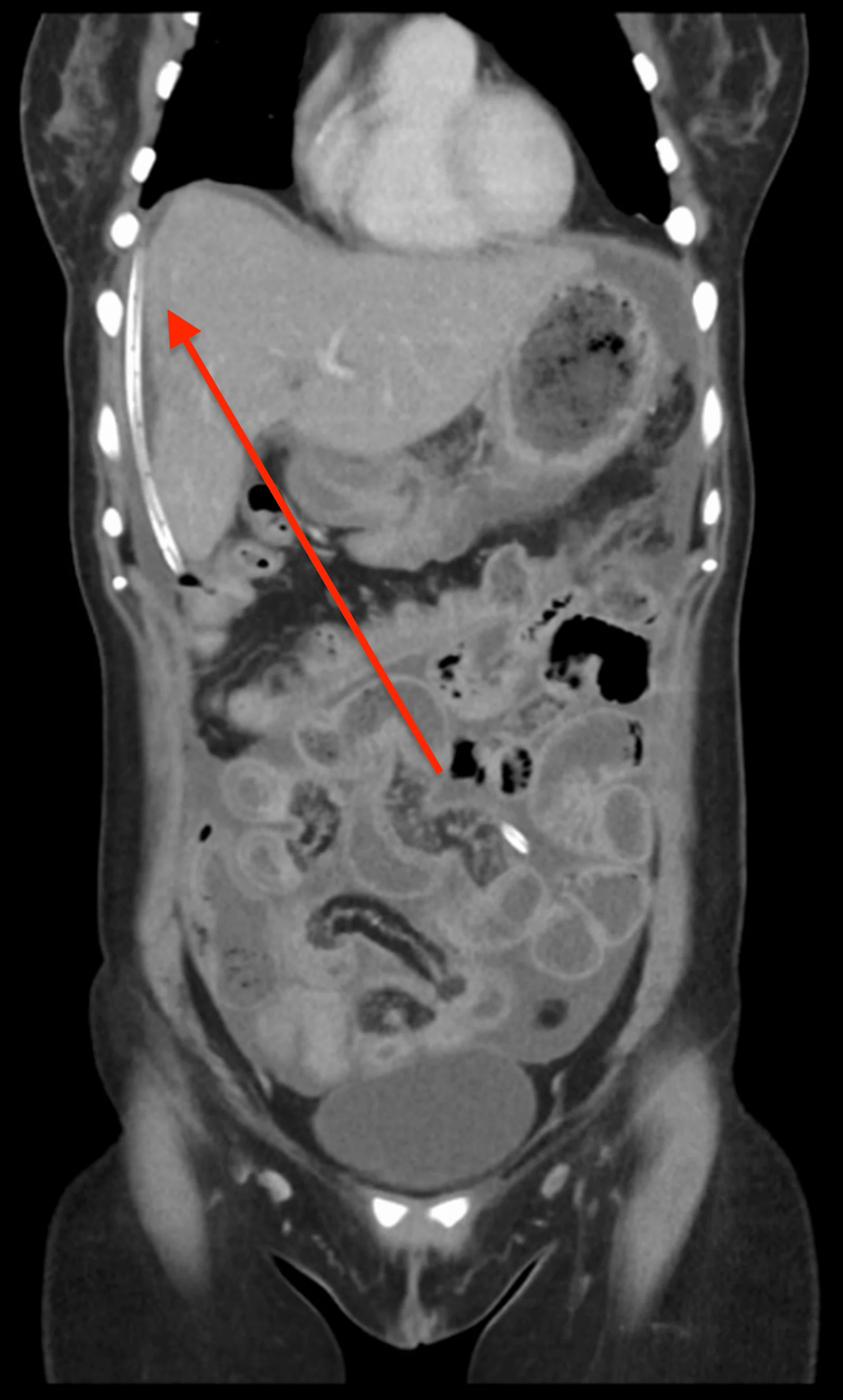
Computer tomography (coronal view) of the abdomen (July 14, 2026) Despite comparatively significantly lower volume of ascites the catheter tip is still located between the thoracic wall and the liver, in contact with the diaphragm.

Case 2

A PTPC was placed in the right lower abdomen of a 71-year-old patient with peritoneal and hepatic metastases from cholangiocellular carcinoma in a palliative care setting. Ten days after placement, the patient presented to the emergency department with a tense, extensive, reddened swelling over the entire right flank, extending from the axilla to the iliac crest. Clinical examination revealed a spongy-elastic swelling with pitting edema, but without signs of systemic inflammation (afebrile, stable vital signs, white blood cell count: 7.6x10^9^/L; C-reactive protein [CRP] 42.1mg/L; of note, CRP levels were elevated weeks prior to PTPC, most likely in context of the underlying malignant disease). Computed tomography showed the catheter in its correct position and revealed an extensive fluid collection, partly epifascial and partly subfascial, extending between the right-sided oblique abdominal muscles on the right, extending across the entire right thoracic and abdominal walls with a craniocaudal extent of nearly 50 cm (Figures 3, 4). In the preceding days, only small amounts of ascites had been drained because of circulatory instability. The findings suggested that ascites was leaking through the peritoneal entry site of the catheter, presumably as a result of increased intra-abdominal pressure. Accordingly, ascites was drained repeatedly in small volumes of 100-200 mL (to avoid circulatory problems) via the catheter, which remained in place, and the patient was additionally positioned on the contralateral side. As a result, the fluid accumulation resolved completely (abdominal CT 7.5 weeks after the initial CT was performed for oncological indications, Figure 5).

**Figure 3:**
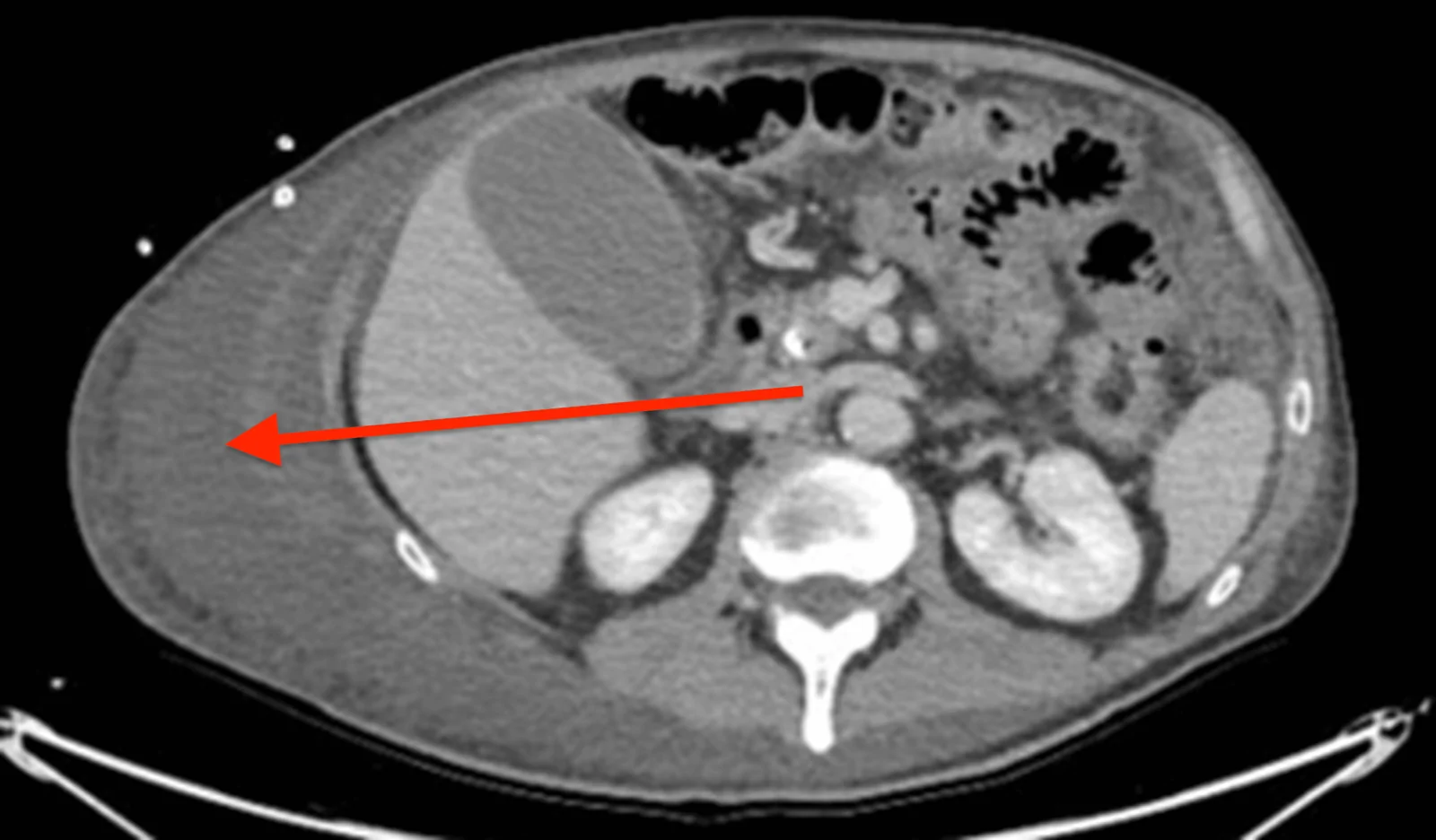
Contrast-enhanced computer tomography (axial view) of the chest and abdomen, venous phase (May 31, 2026) A fluid collection is seen along the right thoracic and abdominal wall, partly epifascial and partly subfascial/intermuscular between the oblique abdominal wall muscles, extending along the entire right thoracic and abdominal wall.

**Figure 4:**
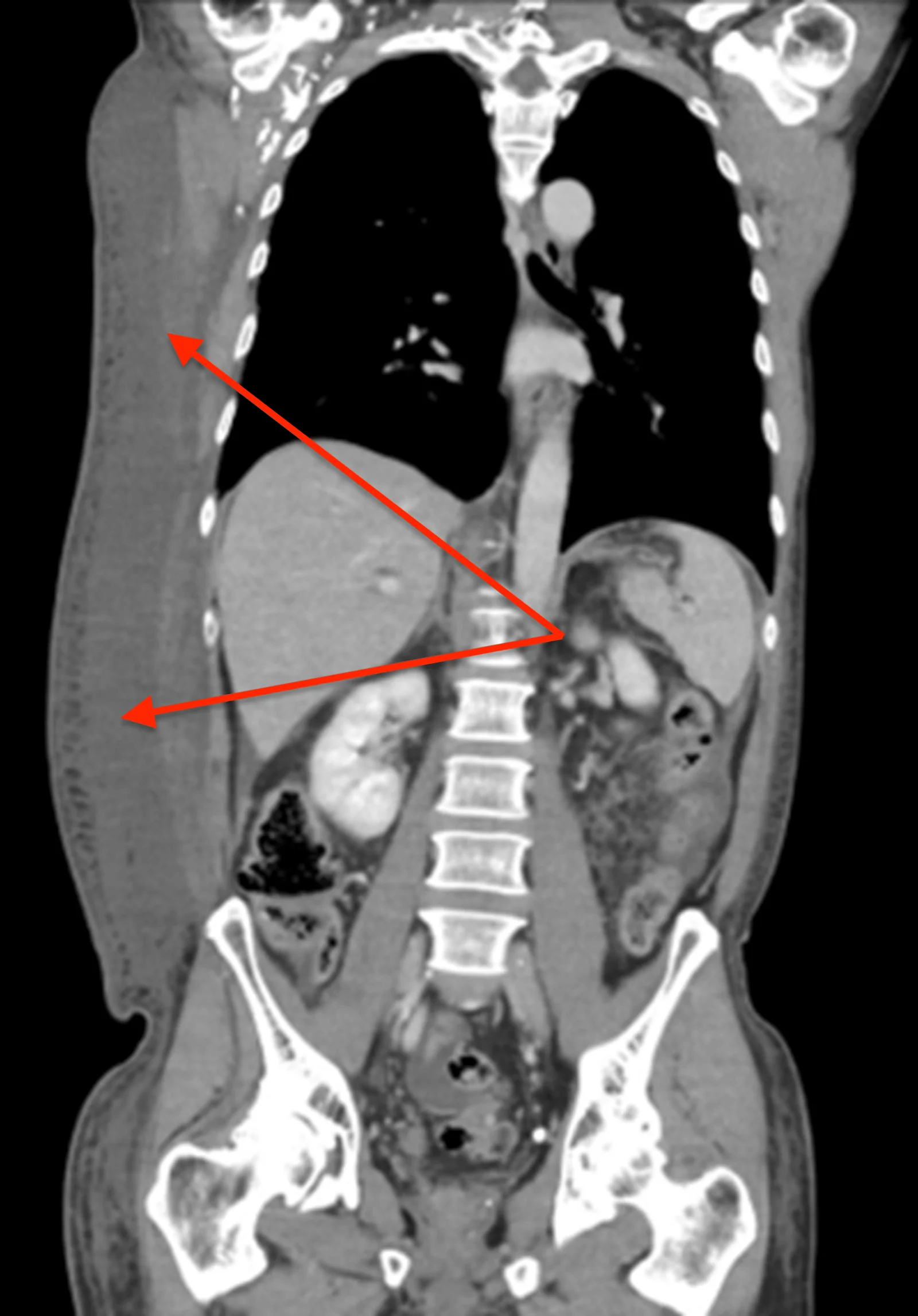
Contrast-enhanced computer tomography (coronal view) of the chest and abdomen, venous phase (May 31, 2026) A fluid collection is seen along the right thoracic and abdominal wall, partly epifascial and partly subfascial/intermuscular between the oblique abdominal wall muscles, extending along the entire right thoracic and abdominal wall.

**Figure 5:**
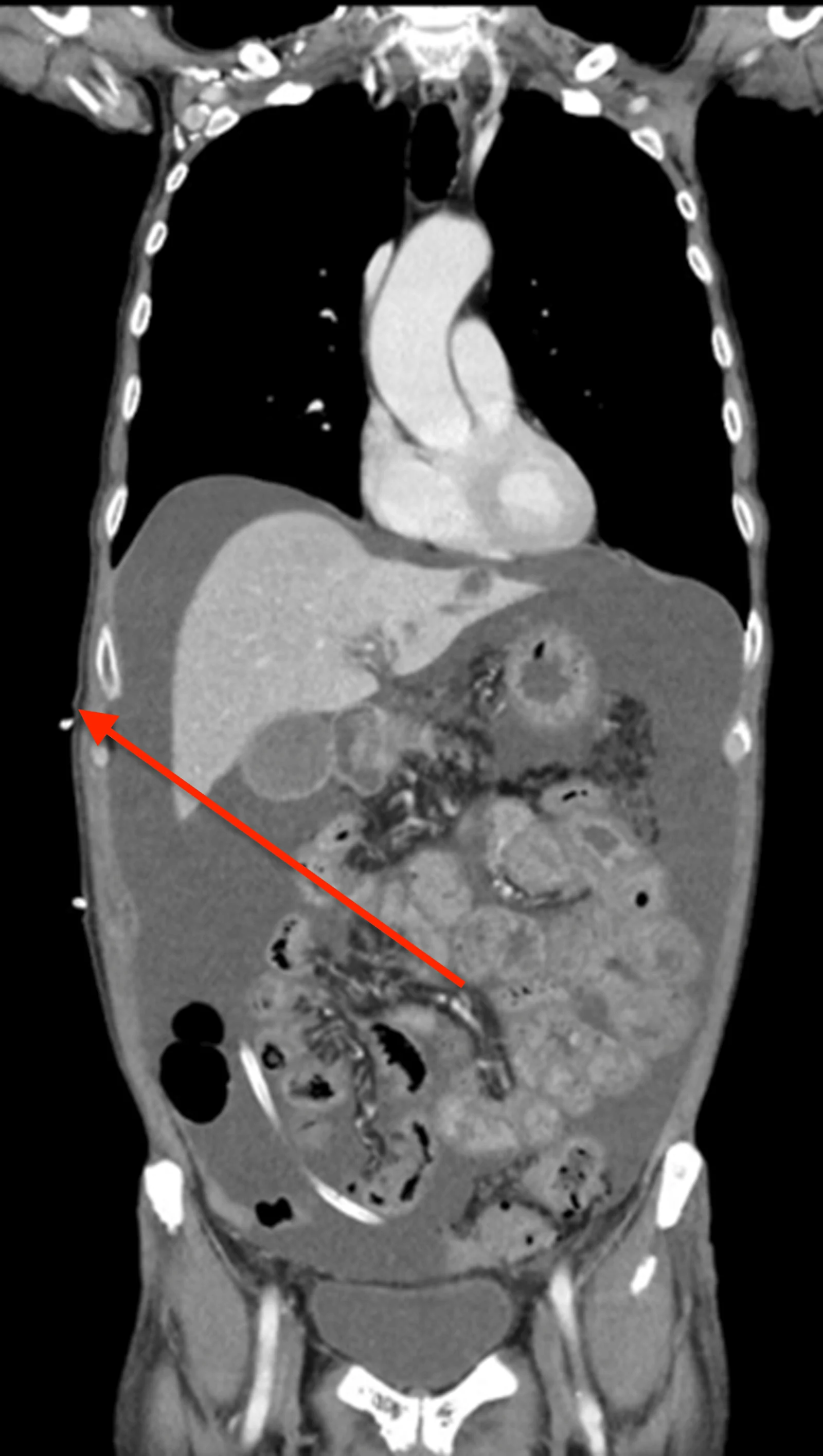
Coronal computer tomography of the chest and abdomen, venous phase (July 23, 2026) With the catheter position remaining unchanged, the previous fluid collection along the right chest and abdominal wall has completely resolved.

## Discussion

PTPCs are generally well tolerated but can occasionally cause unusual and sometimes striking anatomical complications that appear dramatic on imaging but may remain clinically manageable with conservative treatment and do not necessarily require catheter removal.

Overall symptom relief and patient satisfaction with PTPC are high [3], and implantation is technically simple and generally well tolerated. The most common complications are catheter infections and/or peritonitis, usually managed by antibiotics, although refractory cases may require catheter removal. Other known common complications include exit-site cellulitis, pericatheter leakage or obstruction (including fibrin plugs, omental wrap, malposition), acute kidney injury, hyponatremia, and post-drainage hypotension. In addition, in the study by Chan et al., abdominal pain was described in 27% (n=14/51) of all cases [2]. In all patients, pain was controlled by increasing analgesia. In contrast, Knight et al. reported two cases of groin pain eventually leading to the PTPC removal [4].

In general, PTPC removal should be considered when drainage is no longer clinically required, in case of irreversible catheter dysfunction or dislocation, or when catheter-related infection cannot be adequately controlled with antimicrobial therapy. Importantly, a single episode of bacterial peritonitis, superficial cellulitis, or leakage does not necessarily require catheter removal; catheter salvage with appropriate antimicrobial treatment is often feasible [2,5].

We hereby present two uncommon PTPC-related adverse events with distinct clinical and radiological presentations. In case 1, diaphragmatic irritation by the catheter tip resulted in referred right shoulder pain despite correct catheter placement and preserved drainage function. In case 2, ascitic fluid tracked extensively into the abdominal wall, resulting in a striking thoracoabdominal fluid collection without catheter malposition. To our knowledge, these specific presentations have not previously been described with corresponding CT documentation. Importantly, both cases illustrate that unusual or radiologically dramatic findings do not necessarily indicate catheter failure or require catheter removal in clinically stable patients with preserved catheter function.

The right-sided shoulder pain in case 1 can best be explained by direct diaphragmatic irritation, resulting in referred shoulder pain via the phrenic nerve, frequently observed after laparoscopic surgeries with pneumoperitoneum [6,7]. The pain was therefore most likely caused by mechanical irritation of the diaphragm by the catheter tip, which also explains why the symptoms improved with movement. The diagnosis was ultimately made based on CT imaging. At the same time, a complication requiring intervention was ruled out, supporting a conservative approach rather than catheter removal. Why the pain completely resolved despite virtually no change in catheter position or ascites volume remains elusive. No data on a possible neural “habituation” of the diaphragm or the phrenic nerve to a mechanical stimulus exist. A possible explanation could be behavioral changes (slower drainage or changes in position during drainage), as documented for "drain pain" associated with peritoneal dialysis catheters [8-9].

Ascitic fluid leakage after PTPC implantation has been reported in 2-21% of cases, associated with obesity and damaged port membranes [10]. Known risk factors are increased intra-abdominal pressure and impaired tissue healing. Reinglas et al. reported three patients with “significant anasarca and skin edema around the catheter site as a result of a leak between the catheter’s polyester cuff and the skin,” of which two resolved spontaneously, while the third required catheter removal [11]. Their studies did not address the extent of the edema (“around the catheter site”) or the method used to make the diagnosis. Similar phenomena involving edema around the puncture site are also known to occur in peritoneal dialysis [12]. Clinically, these manifest as subcutaneous swelling with a pale discoloration of the skin caused by dialysis fluid seeping into the abdominal wall. In our case (case 2), there was no leakage of ascites from the skin incision. Instead, ascites accumulated in the subcutaneous tissue, causing marked swelling several centimeters in thickness across the entire right flank, accompanied primarily by pain. A CT scan ruled out catheter malposition. The most likely explanation, therefore, was a leak through the peritoneum, presumably caused by high intra-abdominal pressure after only a small amount of ascites had been drained over several days. Consequently, more frequent ascites drainage combined with positioning the patient on the contralateral side resulted in complete resolution of the subcutaneous edema.

## Conclusions

These cases suggest that, provided that the catheter is functioning properly, conservative management with the aim of catheter preservation may be considered in clinically stable patients without evidence of uncontrolled infection or peritonitis, despite potentially dramatic imaging findings or unusual mechanical complications, including catheter-tip-related pain and significant leakage of ascitic fluid into the abdominal wall.
